# Order Matters! Influences of Linear Order on Linguistic Category Learning

**DOI:** 10.1111/cogs.12910

**Published:** 2020-10-30

**Authors:** Dorothée B. Hoppe, Jacolien van Rij, Petra Hendriks, Michael Ramscar

**Affiliations:** ^1^ Center for Language and Cognition University of Groningen; ^2^ Department of Artificial Intelligence University of Groningen; ^3^ Department of General and Computational Linguistics University of Tübingen

**Keywords:** Discriminative learning, Error‐driven learning, Linguistic categories, Computational simulation, Behavioral experiment, Artificial language learning experiment

## Abstract

Linguistic category learning has been shown to be highly sensitive to linear order, and depending on the task, differentially sensitive to the information provided by preceding category markers (*premarkers*, e.g., gendered articles) or succeeding category markers (*postmarkers*, e.g., gendered suffixes). Given that numerous systems for marking grammatical categories exist in natural languages, it follows that a better understanding of these findings can shed light on the factors underlying this diversity. In two discriminative learning simulations and an artificial language learning experiment, we identify two factors that modulate linear order effects in linguistic category learning: category structure and the level of abstraction in a category hierarchy. Regarding category structure, we find that postmarking brings an advantage for learning category diagnostic stimulus dimensions, an effect not present when categories are non‐confusable. Regarding levels of abstraction, we find that premarking of super‐ordinate categories (e.g., noun class) facilitates learning of subordinate categories (e.g., nouns). We present detailed simulations using a plausible candidate mechanism for the observed effects, along with a comprehensive analysis of linear order effects within an expectation‐based account of learning. Our findings indicate that linguistic category learning is differentially guided by pre‐ and postmarking, and that the influence of each is modulated by the specific characteristics of a given category system.

## 1 Introduction

Natural languages abound with regularities, patterns, and conventions. Indeed, philosophers have long noted that to say language is ruled by convention is something of a platitude (Lewis, 2008). Accordingly, in attempting to understand the conventionalized nature of human communication, linguists have expended a great deal of effort on taxonomizing the regularities and patterns observable in the world's languages into various lexical and grammatical categories (such as word class, case, gender, tense, aspect, mood, etc.) based on their form features, or their distributional characteristics, for example their combination with grammatical markers. Interestingly, the case of grammatical markers highlights a dimension highly important for the analysis of regularities in language: linear order. In the case of noun gender, for example, gender markers can either precede the noun (*premarking*, e.g., gendered articles in German: *das* Kind, or noun class prefixes in Swahili: *m*toto), follow the noun (*postmarking*, e.g., noun suffixes in Russian: kartin*a*), or even occupy both positions (e.g., gendered articles and relative pronouns in German: *das* Kind, *das* hier ist). According to typological analyses, postmarking is the most frequent grammatical marking pattern in languages across the world (irrespective of whether the markers are bound morphemes, e.g., Hawkins & Gilligan, 1988, or free morphemes, Bybee, Pagliuca, & Perkins, 1990). This observation has triggered a considerable debate about whether and how the linear order in which categories are marked makes a difference to language processing, to language production, or—as we will investigate here—to language learning.

Previous work on marking order and learning has mainly focused on the advantage of postmarkers for learning grammatical categories. One suggested explanation for this *postmarking advantage* is that postmarkers are perceptually more salient than premarkers (based, e.g., on the observation of final syllable lengthening in French, English, and Russian, Vaissière, 1983; and the rare omission of word‐final unstressed syllables by children, Slobin, 1973; Snow, 1998), and that this promotes learning in general. However, a recent theoretical account suggests that premarkers and postmarkers serve different functions regarding learning and informativity within category systems in language (Ramscar, 2013).

This proposal of separate functions of pre‐ and postmarking stems from the assumption that language learning is based on a mechanism of adjusting learners' expectations (i.e., that learning is *expectation‐based*). Upon hearing the noun stem *kartin‐* (painting) a speaker of Russian will, for example, expect a specific postmarker, the feminine noun ending *‐a*. However, while words can be used to predict a following postmarker, the relation is reversed with premarkers: They predict the words following them. Upon hearing the German neuter article *das*, for example, a listener will expect to hear a neuter noun, as opposed to expecting any noun. These two examples illustrate that due to their differing *linear order relations*, premarkers and postmarkers stand in different *predictive relations* to the words that they are associated with in the grammar. From this expectation‐based learning perspective, it has thus been proposed that premarkers and postmarkers may have different influences on language processing and learning.

The current study investigates how linear order interacts with the structure and level of abstraction of categories in language learning. Although previous work has investigated the different functions of premarking and postmarking, offering evidence in support of an expectation‐based learning account, the vast diversity and intricate hierarchies of categories in natural languages call for further exploration of this phenomenon. Our aim here is to provide a more complete picture of the effects of linear order on language learning by testing the generalizability of linear order effects to different kinds of category systems, and to clarify the kind of processes that lead to these effects. In the remainder of this section, we begin by reviewing expectation‐based learning theory and evidence addressing how linear order affects learning categories in language, in both firs and second language learning situations, before explaining the rationale behind the present study, which was specifically set in a second language learning context.

### 1.1 An expectation‐based learning explanation of the postmarking advantage

The expectation‐based learning account largely accords with accounts based on salience in predicting a postmarking advantage in category learning. A crucial difference, however, is the wider scope of the expectation‐based learning account as it can potentially provide an explanation for the general function of categories in language and for the processes that underlie category learning.

From an expectation‐based learning perspective, category learning is best characterized as a discrimination problem, simply because computationally, learning from prediction is a discriminative learning process based on prediction‐error minimization (Ng & Jordan, 2002; Ramscar, Yarlett, Dye, Denny, & Thorpe, 2010). Seen from this perspective, the aim of category learning is to find out which item features are most relevant to discriminate one category from another rather than clustering items into categories according to similarity. Support for this idea comes from observations showing that many common categories cannot be defined in terms of shared definitive features, which contradicts the idea of clustering by similarity. For example, people easily learn semantic categories such as “fish” that include category members that do not share seemingly defining features (e.g., mud skippers are fish that can live outside of water) and exclude items that do share common features (e.g., dolphins are mammals but look like fish). Another observation that mitigates against the idea of similarity within categories is that there are many categories, including those typically associated by grammatical gender, which comprise items that do not share any features. German gender, for example, has initially been thought to be a mere evolutionary artifact, because its structure has appeared to be so random to many observers. Furthermore, evidence suggests that seemingly unrelated items can be learned to be members of common categories (Ramscar, 2013). Accordingly, it has been suggested that these various findings do not support the idea that categories cluster together things with somehow inherently similar characteristics, but rather that categories are sets of items that share a common label (Ramscar & Port, 2019). This view proposes that learning to associate a set of items with a category label is not merely a process of recognizing similarities, but rather is a process of increasing discrimination between items that share a given label and those that do not (see also Rescorla, 1988).

Expectation‐based (or *error‐driven*) learning models have been both influential and widely employed in psycholinguistic research and in psychology in general (e.g., Aizenberg, Aizenberg, & Vandewalle, 2013; Dayan & Daw, 2008; Hannun et al., 2014; Rescorla & Wagner, 1972; Rumelhart & McClelland, 1987). Critically, all error‐driven learning models implement discriminative learning algorithms (Ng & Jordan, 2002; Ramscar et al., 2010). A first, basic assumption of a *discriminative* account of category learning is that this kind of learning does not simply involve the tracking of contingencies between stimuli (e.g., between animal features and a species label, or between noun features and a gender marker) but that it estimates how much *information* one item or event, a *cue*, can provide about another item or event, an *outcome* (Rescorla, 1988). The aim is to produce an estimate of how informative a cue is for an outcome, and this is achieved by a learning mechanism that uses the informativity of cues to gradually reduce its uncertainty about the likelihood of an outcome. This process not only *associates* informative cues with an outcome but it also *dissociates* uninformative cues from that outcome. A second, basic assumption at the core of error‐driven learning rules is that cues are competing with each other for informativity, which is a demising resource as learning progresses. The interplay of association, dissociation, and cue competition yields a process that is guided by the informativity rather than the frequency of cues. A critical function of this mechanism is to dissociate irrelevant features which are nevertheless shared between many items in a category, for example that fish live in water but are still not most relevant for discriminating the category from other categories on the same level of abstraction, for example, fish from mammals.

Third, because the discriminative form of learning implemented in expectation‐based models is ultimately determined by prediction‐error, it is asymmetric. Accordingly, learning is not assumed to determine the association between cues and outcomes (*↔*) but rather the association of a cue with an outcome (*→*). Crucially, there is evidence that the asymmetry of learning results in a cue–outcome order effect of learning (or *feature‐label order effect*, Ramscar et al., 2010): Learning potentially differs whenever the order of two items or events, for example, first seeing a fish and then hearing someone say “fish”, is reversed. In a task in which learners had to learn the names of novel object categories, Ramscar et al. (2010) found that learning was facilitated whenever object images preceded category labels during training, as compared to when object images were shown after the category labels. This suggests that we need to consider two possible learning situations for a categorization task: Either the category labels follow the items^1^ that have to be categorized, or the category labels precede the items.

If we transfer these expectation‐based learning principles to grammatical category learning, which is the focus of this article, we can differentiate between two kinds of learning situations: *premarking* and *postmarking situations*. In a premarking situation, the grammatical marker can be operationalized as cue to the features of the following word. In a postmarking situation, the grammatical marker can be interpreted as an outcome cued by preceding word features.

**Fig. 1:**
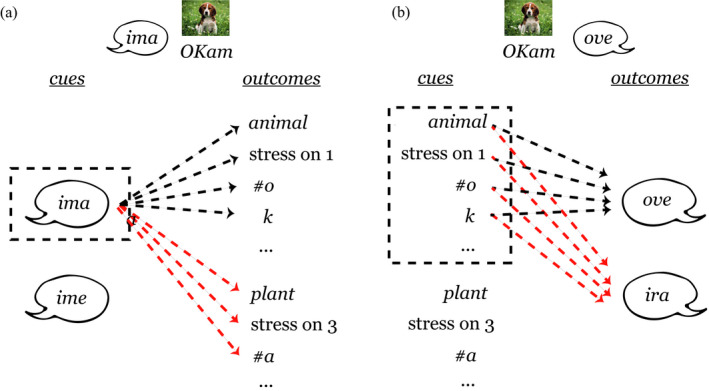
Illustration of the difference between learning in (a) a premarking situation and (b) a postmarking situation. In this example, based on the materials used in the simulations and behavioral experiment (see Table 2), a learner either needs to associate noun class markers (e.g., *ima*) with a noun and its form features (e.g., stress or phones) and semantic features (e.g., *animal*) or the other way around. In the divergent premarking situation (a), there is little cue competition (dashed black box). In the postmarking situation (b), the relation between cues and outcomes is convergent, which leads to many cues competing with each other (dashed black box). Moreover, the pattern of association (black dashed lines) and dissociation (red dashed lines) is not mirrored between (a) and (b), which shows the asymmetry of the discriminative learning mechanism. Note that capitals mark syllable stress.

Fig.1 illustrates how marking order could affect learning of noun class categories depending on their specific form and semantic features. An analysis of the contrasting premarking and postmarking situations from a discriminative learning perspective suggests that they can give rise to different learning dynamics (and learning outcomes), although the basic mechanisms—association, dissociation, and cue competition—are active in both marking orders. In a postmarking situation, cue sets are larger and potentially overlapping, and cues and outcomes are in a *convergent* relation (Osgood, 1949, see Fig. 1b). Therefore, more cues compete for an outcome which makes cue competition more effective in postmarking. This leads to a process which is driven mainly by the informativity of features for a category marker (e.g., Ramscar et al., 2010). In contrast, in premarking situations cues and outcomes are usually in a *divergent* relation with more outcomes than cues (see Fig. 1a). In such a situation, noun features do not compete for the labels as cues but as outcomes. Outcome competition is more driven by frequency than by informativity, and this leads to the learning of conditional probabilities of features given a category marker (Hoppe, Hendriks, Ramscar, & van Rij, 2020; Ramscar, 2013).

A number of findings in linguistics show indeed an advantage of postmarking over premarking in category learning. Evidence from language acquisition suggests that children learn suffixes faster than prefixes (Clark, 2001; Kuczaj, 1979) and in particular, that inflectional systems are learned earlier when they are encoded by suffixes than when they are encoded by preceding markers (Slobin, 1973). Further support for a postmarking advantage is provided by a number of recent artificial language learning studies. For example, St Clair, Monaghan, and Ramscar (2009) demonstrated that participants were significantly better at recognizing previously trained compatible and incompatible affix–word combinations when those affixes were suffixes rather than prefixes; Ramscar (2013) found that words that shared a suffix were rated more similar to each other than words that shared a prefix; and Nixon (2020) showed that English learners were better at learning to discriminate tonal syllables from Southern Min Chinese when category markers (in this case, geometrical shapes) followed the training syllables than when they preceded them.

Thus, in the context of an expectation‐based learning account, the postmarking advantage follows from the cue competition in a convergent learning situation. Next, we will explore whether and how this postmarking advantage extends to differently structured categories and categories at different levels of abstraction in a category hierarchy, an investigation which will bring us also to the function of premarking in category learning.

### 1.2 Category structure and the postmarking advantage

The first aim of the present study is to investigate whether the postmarking advantage generalizes to differently structured categories. Regularities in language differ highly in their structural characteristics, for example, how informative item features are for a category (cue validity, Rosch, Mervis, Gray, Johnson, & Boyes‐Braem, 1976; feature diagnosticity, Minda & Smith, 2001), the ratio of within‐category similarity and between‐category similarity (structural ratio, Minda & Smith, 2001), or the number of bits that are needed to code a category (entropy, Shannon, 1948). Not surprisingly, these factors have been found to affect how easy it is to learn a specific category system (e.g., Lafond, Lacouture, & Mineau, 2007; Reeder, Newport, & Aslin, 2013).

We suggest that in expectation‐based learning theory, the amount of overlap between categories determines the need for postmarking in contrast to premarking: The postmarking advantage for category discrimination might be reduced when categories share fewer overlapping features. In experiments in which a postmarking advantage has been observed, category systems showed a high amount of overlap, for example, highly frequent features that are shared across categories and that are therefore uninformative for category discrimination (Nixon, 2020; Ramscar, Dye, Gustafson, & Klein, 2013; Ramscar, Dye, Popick, & O'Donnell‐McCarthy, 2011; Ramscar et al., 2010). In these cases, cue competition during postmarking helps to dissociate such frequent uninformative features. In contrast, more distinct categories elicit less cue competition and, as a consequence, the dissociation of uninformative cues is reduced. In such situations, the resulting learning relation with a marker should be more symmetric than in Fig.1, leading to a less pronounced asymmetry effect between marking orders.

It is important to note here that defining the amount of overlap between categories is not a trivial task given that categories are not inherently grounded in objective properties of the world (Ramscar & Port, 2019). Assuming that categories are rather functional units in a communication system, a specific category representation is more likely determined by the whole system of category contrasts acquired by a specific learner. This can, for example, be illustrated with the learning of new phonological categories in a second language: While to a native speaker of a tone language phonemes differing only in tone appear completely distinct, native speakers of English can only master the discrimination of tones by relearning acoustic cues as informative which have been unlearned under a predominant exposure to English (as in Nixon, 2020). Indeed, direct evidence suggests that which cues learners rely on to discriminate categories is determined by learning history (Arnon & Ramscar, 2012; Culbertson, Gagliardi, & Smith, 2017; Ramscar et al., 2013). Hence, with “overlap” between categories we, here, refer to the perceived amount of overlapping (i.e., confusable) features between previously learned category representations.

From an expectation‐based learning perspective, we do not expect that the postmarking advantage generalizes to any and every type of category learning situation. In particular, we hypothesize that the more categories overlap (such that members of different categories are more confusable), the stronger the advantage that postmarking brings for category discrimination. As a consequence, we predict that categories already learned to be distinct will subsequently not profit more from postmarking than from premarking. Concerning the underlying learning mechanism, such a finding would corroborate the idea that category discrimination is mainly a process of dissociating overlapping and therefore confusable features in search for the features that are most informative for the discrimination.

### 1.3 The premarking advantage

In mastering a language, learners are not only confronted with different category structures, they are simultaneously required to learn category contrasts at various levels of abstraction. These levels of abstraction in a category hierarchy can be characterized in terms of their inclusiveness (meaning how many specific entities a category includes, Rosch et al., 1976). To examine linear order effects across the full diversity of category systems, we will further investigate how marking order affects category learning at different levels of abstraction.

Thus far, we have seen that dissociation of features that are uninformative for a category contrast clearly facilitates categorization. However, for other tasks, this kind of information loss can become detrimental: For example, while in learning to discriminate fish from mammals, *living in water* is not always an informative feature, it is in fact useful to discriminate a sardine from a mud skipper. Note that in this example, the contrast between the type of fish is on a lower, more fine‐grained level of abstraction than the contrast between types of species. Similarly, we might expect that the features that are relevant to discriminate feminine from masculine German nouns (in this case, the *super‐ordinate category contrast*) differ from the features that are relevant to discriminate single feminine nouns from each other (the *subordinate category contrast*). This suggests that there is a trade‐off between optimally discriminating super‐ordinate and subordinate categories, due to the information loss which is necessary for the discrimination process (Dye & Ramscar, 2009).

This trade‐off suggests further that knowledge gained on one level of abstraction does not always generalize to other levels of abstraction. In particular the facilitation of postmarking on super‐ordinate category levels cannot be transferred to subordinate levels. This idea is supported by the findings of Ramscar (2013), who performed an artificial language learning task comparing noun learning and noun category learning. In this study, participants were first trained to associate invented nouns with random known objects, the subordinate category contrast. After that, they heard sentences consisting of phrases containing the noun labels paired with different markers signaling a super‐ordinate category contrast. A subsequent similarity test confirmed that postmarkers helped super‐ordinate category discrimination: Participants rated objects to be more similar to each other when their corresponding nouns shared a postmarker than when they shared a premarker. However, a grammaticality judgment task showed that participants were better at learning the nouns' meanings—here the *sub*ordinate category contrast—when nouns were marked on the *super*‐ordinate category contrast by a premarker and not a postmarker during training.

Results from a study by Arnon and Ramscar (2012) suggest that this effect of improved noun learning after a noun class premarker is indeed due to the presence of premarking and not merely the absence of postmarking. This study investigated a different question, namely, whether the learning of article–noun associations in a second language could be blocked by previous learning of the nouns' meanings, a hypothesis which their findings corroborate. They also observed that learners were significantly better at learning to associate objects with invented nouns when the nouns were preceded by previously learned noun class articles than when they had to learn the object–noun associations without article support. Hence, the previous knowledge of the super‐ordinate noun classes in combination with the articles seemed to have facilitated noun meaning discrimination.

Here, we aim to investigate in detail what processes underlie this *premarking advantage* that super‐ordinate premarkers seem to have on learning subordinate categories. An explanation for the premarking advantage put forward in Ramscar (2013) and Arnon and Ramscar (2012) is that premarkers serve a communicative function in that they reduce uncertainty about following words, by eliminating words that do not belong to the marked category from the set of possibly following words (Dye, Milin, Futrell, & Ramscar, 2017). A basic assumption of the expectation‐based learning account is that communication has the general aim of reducing uncertainty, such as for example, a listener's uncertainty about the intention of a speaker. Seen from this perspective, different levels of abstraction in a category hierarchy would coincide with different levels of uncertainty reduction: On the level of noun classes, for example, uncertainty is reduced from all possible nouns to the subset of nouns from one class. Learning nouns in such a reduced set seems to be advantageous as compared to learning them in the full set of possible nouns. However, why this is the case is not clear, yet. To investigate this question, we will therefore simulate noun learning within and across noun classes with a discriminative learning model using error‐driven learning and then seek to confirm this effect in a behavioral experiment.

### 1.4 The present study

The present study investigates how linear order interacts with the structure and level of abstraction of categories in language learning. While there is evidence that the various factors introduced so far—linear order, category structure, and levels of abstraction—all influence learning of linguistic categories, thus far these effects have been studied in isolation. In what follows, we will seek to examine the degree to which these factors interact and/or complement one another in a second language learning situation.

By investigating category structure and level of abstraction, we want to link the discussion about linear order effects with the discussion about the functional role of category markers and hope to contribute also, indirectly, to a better understanding of the functional role of categories in language. In particular, we assume that categories in language serve their function as part of a system of communication. From this perspective, postmarkers serve to help in the discrimination of relevant category contrasts, whereas premarkers serve to guide the process of uncertainty reduction about an intended message and at the same time focus the discrimination problem to subordinate levels of abstraction in a category hierarchy.

In Section 2, we will first discuss two simulations of discriminative learning that we implemented to examine how linear marking order affects learning categories with different structures and at different levels of abstraction in an artificial category system. In Section 3, we present the results of an experiment in which adult participants were trained on the same artificial language to test the predictions of the simulations.

## 2 Modeling linear order effects in category learning

To examine how linear marking order affects learning categories with different structures and at different levels of abstraction, we designed an artificial language built around a noun class system that varied in both of these factors. In this section, we present two computational models that simulate how a language learner would acquire this noun class system, from an expectation‐based perspective using error‐driven learning. The first model simulates how premarking and postmarking of noun class affect noun class learning (the super‐ordinate category contrast), whereas the second model simulates how premarking and postmarking of noun class influence noun learning (the subordinate category contrast) within the same artificial language. We will start with presenting the structure of the artificial language.

### 2.1 Artificial language

The artificial language consisted of a differentially structured and hierarchical artificial noun class system. This system was built around two‐ and three‐syllabic imaginary nouns (see Table 1) describing different visualizable real‐life concepts (see Tables 2 and 4). These nouns were then systematically assigned to different noun classes which were either all marked by a specific premarker or by a specific postmarker.

**Table 1:** The training nouns for the simulations and the behavioral experiment

	Noun Class 1	Noun Class 2	Noun Class 3	Noun Class 4	Frequency
Premarker	ima	imo	ime	imi	
Noun	oksham	kanjur	anveal	jajosan	32
	luobar	ennovis	psondew	serim	23
	anhatar	ruis	hatrumir	erkefal	16
	simad	lopranik	kilal	vimeros	11
	nechran	aftong	repis	burbad	8
	kekunam	palneng	tokran	ksoster	6
	kitsogis	tivitkal	istefur	natrul	4
	magril	meromer	merkatim	rutonak	3
Postmarker	ove/ovu	ira/ire	agi/ago	epo/epa	

Note

The vowel alternation of the postmarkers was dependent on the carrier phrases *unta boltohe* (appearing with *ove*, *ira*, *agi*, and *epo*) and *ena dikanhe* (appearing with *ovu*, *ire*, *ago*, and *epa*).

**Table 2:** The four noun classes of the artificial language and their combination of meaning and form category features

			Form Categories		
			Unambiguous		Ambiguous
			Stress on 1	Stress on 2	Stress on 3/4
Meaning Categories	Unambiguous	Animal 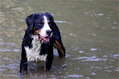	Noun class 1 ***ima*** *X agi* or *imo X* ***ove***	—	—
		Plant 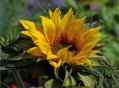	—	—	Noun class 2 ***imo*** *X agi* or *imo X* ***ira***
	Ambiguous	Random 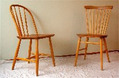	—	Noun class 3 ***ime*** *X agi* or *imo X* ***agi***	Noun class 4 ***imi*** *X agi* or *imo X* ***epa***

Note

In the premarking variant, the unspecific postmarker *agi* was added to all nouns, in the postmarking variant, the unspecific premarker *imo*. Moreover, ambiguous categories are shared with another noun class, while unambiguous categories only appear in one noun class.

We manipulated marking order in that a noun always followed a premarker and preceded a postmarker. Two different marking variants determined whether the premarker or the postmarker aligned with the four noun classes or not. In the *premarking variant*, four premarkers, *ima*, *imo*, *ime*, and *imi,* were consistent with their noun class and one unspecific postmarker, *agi*, was used for all nouns. In the *postmarking variant*, one premarker, *imo*, appeared with all nouns and four postmarkers, *ovu*, *ira*, *agi*, and *epo,* were consistent with their noun class. The combinations of markers and nouns were then embedded into a context by a sentence‐initial carrier phrase (*ena dikanhe*, which could mean “he is talking about …”, or *unta boltohe*, which could mean “he is dreaming of …”).

In both variants, the last vowel of each postmarker was dependent on the carrier phrase, for example, *ovu* would turn into *ove* for carrier phrase two. An example sentence of the premarking variant is given in (1).

**Table:** 

(1)	Unta boltohe	ima	OKsham‐	agi.
	Carrier phrase1	premarker1	“dog/dogs”	unspecific postmarker
	He is dreaming of dogs.			

To address our first question of how category structure interacts with linear marking order, the nouns and their associated images were manipulated on two dimensions; on their form by assigning them to one of three syllable stress categories (*form categories*: stress on first, second, or last^2^ syllable), and on their meaning by assigning them to one of three different semantic categories (*meaning categories*: animals, plants, or random objects). The noun *oksham* in Example sentence (1) from Noun class 1 was, for example, stressed on the first syllable (capitals mark the stressed syllable) and used to refer to dogs (the artificial language was not specific about number). Note that during the recording of stimuli for the behavioral experiment, postmarkers were read as suffixes attached to the nouns. For nouns from Noun class 2 and 4, stress therefore fell on the postmarker.

We assumed that the form categories were perceived as more overlapping than the semantic categories based on the differing learning context and an adult learner's previous knowledge about the two category types. Both the meaning and form categories we used are contrastive—thus, already learned—categories in the L1 of the Dutch learners.^3^ However, the meaning features were integrated in images showing already familiar objects in a familiar context, whereas the stress features were part of a very complex speech stream that consisted of many unknown sound combinations. Thus, the familiar context in the images should facilitate the transfer of the meaning category knowledge, but the unfamiliar language context should hinder such a transfer of category knowledge for the form categories. We therefore assumed that the meaning categories were perceived as already learned and therefore distinct categories, while the form categories still had to be formed in this new context and should be perceived as overlapping categories.

The form and meaning categories were then combined pairwise to form three noun classes. To increase the complexity of our artificial noun class paradigm and to make it more comparable to real noun class paradigms, we induced marking ambiguity by adding a fourth marked noun class category that shared the stress category from one and the meaning category from another noun class. In this way, we simulated ambiguity of some of the linguistic features, for example, as in marking syncretisms in the German case and gender system. Overall, this yielded four noun classes with all levels of ambiguity (1: completely unambiguous, 2: ambiguous in distinct feature set, 3: ambiguous in overlapping feature set, 4: completely ambiguous) as illustrated in Table 2. In addition, the frequency of nouns within each noun class followed an exponential (or strictly speaking a geometric) distribution to provide a distribution of words within categories which matches natural word distributions (Guo, Chen, & Wang, 2011; Kim & Park, 2005; Linke & Ramscar, 2020; Ramscar, 2020).

To address our second question of how linear marking order interacts with different levels of abstraction, the category system of this artificial language has two levels of abstraction. On the *noun level* (subordinate category), nouns categorize specific meanings (e.g., the set of dogs or the set of cats) and on the *noun class level* (super‐ordinate category), the noun classes categorize nouns. This structure allows us to compare the effects of linear order on learning the noun classes and the specific noun meanings. Crucially, only the order of the noun class marking was manipulated while the order of nouns and images (meanings) was kept constant (in the behavioral experiment nouns and images were presented at the same time). Another important point is that the meaning categories (i.e., plants and animals) are familiar and therefore non‐confusable categories for adult learners. Therefore, we assume that noun class *premarking* reduces the uncertainty about the possible meanings of a noun. For example, we assume that after hearing *ima* (i.e., the premarker for the animal noun class, see Table 2), the listener will learn to expect an animal as possible outcome for the upcoming noun. Furthermore, it is important to note that features discriminating nouns within a noun class are potentially overlapping between categories, because the nouns were pseudorandomly assigned to noun classes, leaving nouns with similar characteristics, as, for example, identical starting sounds, distributed over the noun classes (see Table 1).

This artificial noun class system offers two different category structures, the distinct meaning categories and the overlapping form categories, and two levels of abstraction, noun categories on the subordinate level and noun class categories on the super‐ordinate level. Both computational models (and later our participants in the behavioral experiment in Section 3) were trained and tested with either noun class premarking or noun class postmarking on the different category contrasts implemented in the artificial category system.

### 2.2 Simulation 1: Linear order and category structure

We begin this investigation of order effects with a simulation of discriminative learning using an error‐driven learning rule to investigate the effect of linear marking order and its interaction with category structure, our first main question. We implemented two variants of the simulation, one in which noun class was marked by premarkers and one in which it was marked by postmarkers. The task of the model was to categorize the artificial nouns into the noun classes that were defined by the distinct meaning categories and the overlapping form categories. During training, the respective marking variant of the model was simultaneously presented with both noun class dimensions, form and meaning. During testing, we separated the feature dimensions, to analyze how these features contributed to the categorization. We hypothesized that both premarking and postmarking use the distinct meaning features to determine the noun class, but that postmarking is more successful than premarking at categorizing nouns using the overlapping form features.

#### 2.2.1 Error‐driven learning

The error‐driven learning rule we use in our simulations is the delta rule originally defined by Widrow and Hoff (1960; which is also a simplified version of the learning rule by Rescorla & Wagner, 1972, see, e.g., Stone, 1987). This simple form of error‐driven learning assumes that cues and outcomes are connected in a fully connected two‐layer network. The association strength or *weight* from cues to outcomes is computed over discrete training trials, saving a weight matrix for every point in time. The weight matrix *V* between cues *i* and outcomes *j* at time *t* + 1 is updated as follows:

(1) $$V_{\mathrm{ij}}^{t+1}=V_{\mathrm{ij}}^{t}+\Delta V_{\mathrm{ij}}^{t}$$

The weight difference $\Delta V_{\mathrm{ij}}^{t}$ at every time step *t* is thereby calculated depending on one of three possible learning situations:

(2) $$\Delta V_{\mathrm{ij}}^{t}=\left\{\begin{array}{cc} 0, & \text{cue}\;i\;\text{absent}\\ \eta (1‐{\text{act}}^{t}\left(j\right)), & \text{cue}\;i\;\text{and}\;\text{outcome}\;j\;\text{present}\\ \eta (0‐{\text{act}}^{t}\left(j\right)), & \text{cue}\;i\;\text{present but outcome}\;j\;\text{absent}. \end{array}\right.$$

In this discriminative learning process, both positive and negative evidence is considered. In the case of positive evidence (second case of Eq. 2), when a cue appears with an outcome, the weight will be increased relative to the difference of the activation act*^t^*(*j*) of outcome *j* given the currently present cues and the maximally possible outcome activation of 1. The outcome activation is calculated as follows with *v*(*i*, *j*) determining the weight between a cue *i* and outcome *j* at time *t*:

(3) $${\text{act}}^{t}(j)=\sum_{x\in \text{cues}(t)}v^{t}(x,j)$$

In the case of negative evidence (third case of Eq. 2), when an outcome does not appear after a cue, the outcome activation will be subtracted from 0 so that the summed cue values in the outcome activation act*^t^*(*j*) will have a negative impact. For all absent cues, there will be no change in weight to any outcome. The learning parameter *η* determines the learning rate and is typically set to the value 0.01.

The characteristic behavior of discriminative learning arises in this error‐driven learning network due to three factors. First, the processing of negative evidence leads to dissociation of cues with a high background rate, which means that these cues occur frequently in general, but do not reliably predict a specific outcome. Second, weights are always updated relative to the sum of the weights of all present cues to an outcome (i.e., the activation act*^t^*(*j*)); if an outcome is already highly predicted by other cues, a new predictive cue will have more difficulties to approach a high weight and will only do so if it proves to be more predictive over a period of time. Third, the possible increase in weights is restricted by the maximal cue value of 1, and it is inversely related to the activation, which makes the network very flexible. For example, a set of low‐frequency cues can quickly become highly predictive, because their low activation value results in a large increase in weight. Overall, the combination of these three factors results in cues *competing* for specific outcomes such that weights will approach the predictive value of a cue for an outcome irrespective of cue frequency. Crucially, this mechanism is asymmetric and outcomes compete differently than cues: When outcomes compete for cues, weights will mirror the conditional probabilities of the outcomes given a cue (see Ramscar, 2013; Ramscar et al., 2010, for empirical support of these model predictions).

Both simulations employ a version of the learning rule specified in Eqs. (1), (2), (3) implemented in R (R Core Team, 2019) using the *edl* package (van Rij & Hoppe, 2020) and the *ndl* package (Arppe et al., 2018). The scripts are available in the Supporting Information.^4^

#### 2.2.2 Training

The premarking and postmarking models were both trained on the same representations, which were created to capture all of the features of the artificial language. The representations consisted of the artificial nouns (see Table 1) to which we added representations of the meaning and form features as well as the specific noun meanings. Given that in the behavioral experiment (presented in Section 3), nouns were presented acoustically, the nouns were split up into uniphones that were marked for word beginning and ending (e.g., #o, k, ∫, a, m#). Our assumption was that the meaning categories would be perceived as distinct. Therefore, we represented the meaning features as three *distinct feature sets* consisting of a single feature each (D1meaning, D2meaning, D3meaning) which corresponded to the three semantic categories in the artificial language (*animal*, *plant*, or *random*). On the other hand, we assumed the form features to be perceived as overlapping. Therefore, we represented these as three *partly overlapping feature sets*, consisting each of one category‐distinct feature (D1form, D2form, D3form) and two features that were shared with one of the other categories (O1form, O2form, O3form) as shown in Table 3. Although these features were abstract representations, the category‐distinct features could be interpreted to correspond to the position of the stressed syllable in a stress pattern and the non‐distinct features to the positions of the unstressed syllables, which are partly shared between different stress patterns. For example, the abstract form feature set {D1form, O1form, O2form} of noun class 1 then corresponds to the features {*1st syllable stressed*, *2nd syllable unstressed*, *3rd syllable unstressed*}. Note that this translation of abstract features into stress features of the artificial language does not consider the variation in word stem length (i.e., that stems could have two or three syllables) in the artificial language but only considers the short two‐syllable word stems with a postmarker suffix. Every noun instance was then defined by a combination of a distinct meaning feature set, a partly overlapping form feature set, noun uniphones, and noun meaning (e.g., {D1meaning, D1form, O1form, O2form, #o, k, ∫, a, m#, dog}).

**Table 3:** The category system of Simulations 1 and 2 and its combination of distinct feature sets (meaning categories) and partly overlapping feature sets (form categories)

			Partly Overlapping Feature Sets		
			Unambiguous		Ambiguous
			{D1form, O1form, O2form}	{D2form, O2form, O3form}	{D3form, O1form, O3form}
Distinct Feature Sets	Unambiguous	{D1meaning}	Noun class 1 marker1 X or X marker1	—	—
		{D2meaning}	—	—	Noun class 2 marker2 X or X marker2
	Ambiguous	{D3meaning}	—	Noun class 3 marker3 X or X marker3	Noun class 4 marker4 X or X marker4

The two models were then trained on these feature sets in combination with a noun class marker (marker1, marker2, marker3) according to the noun category paradigm of the artificial language.^5^ In the *premarking model*, noun class markers were given as cues to the model and the noun features were given as outcomes such that the model’s task was to predict a noun from a marker, for example:

{marker1, constant} → {D1meaning, D1form, O1form, O2form, #o, k, ∫, a, m#, dog}

In the *postmarking model*, noun features were given as cues to the model and noun class markers as outcomes such that the model's task was to predict a marker from a noun, for example:

{D1meaning, D1form, O1form, O2form, #o, k, ∫, a, m#, dog, constant} → {marker1}

We, furthermore, added a constant cue (constant) to every training trial, which accounts for additional constant background information that, for example, a learner brings to a learning situation. Typically, weights in an error‐driven learning model asymptote at a level that minimizes the sum‐of‐squares prediction error for a set of outcomes over a set of observed cue sets. The presence of the constant cue serves a function that can be linked to that of the intercept term in a regression model, in that it serves to ensure that the mean of these errors is zero. In addition, this cue ensures a minimal amount of cue competition in the premarking condition, as learning situations entirely lacking cue competition are highly unrealistic.

#### 2.2.3 Model evaluation

First, we inspected the weight development over time to get a closer understanding of the dynamics during premarking and postmarking learning. After the model had been trained to asymptote, we inspected the model's ability to discriminate between the categories based only on the distinct or the overlapping dimensions, depending on whether it had been trained with premarking or postmarking.

Second, to be able to make predictions about the categorization performance of a learner after premarking and postmarking training, we calculated the probability with which the model would predict the correct postmarker from a feature set or the correct feature set from a premarker. Probability of making a correct choice was calculated based on the models' outcome activations (see Eq. 3).

One problematic point in comparing categorization performance after premarking and after postmarking is in our case that the choice baselines differ between the training conditions. While in the premarking model, the premarker cue makes predictions about three possible outcomes (noun feature sets), resulting in a baseline of 1/3, in the postmarking model, a cue set consisting of the noun features makes predictions about four possible outcomes (postmarkers), resulting in a baseline of 1/4. To circumvent this issue, we calculated the probabilities of choosing the correct outcome set in the premarking and the postmarking model compared to each of the other possible outcome sets and then defined the accuracy of choosing this outcome set as the mean over the probabilities of these binary choices. This resulted in a baseline of 1/2 over all conditions. Probabilities were then calculated according to Luce's choice axiom (Luce, 1959) after applying a rectified linear activation unit (ReLU) to the activation data which set all negative activations to zero. In sum, the probability *P_c_* of choosing the correct outcome (set) *x* in a set of choice alternatives *O*, including competitor outcomes *y* ∈ *C* ⊂ *O*, was calculated as follows:

(4) $$P_{c}\left(x\right)\;=\;\text{mean}\left(\sum_{y\in C}\frac{\text{ReLU}(\text{act}(x))}{\text{ReLU}(\text{act}(x))+\text{ReLU}(\text{act}(y))}\right)$$

For postmarking predictions, the probability of a correct choice was calculated over the activations of a postmarker given a feature set and the constant cue. As due to the ambiguity manipulation, some feature sets correctly predicted two postmarkers (e.g., the overlapping feature set {D3form, O1form, O3form} appeared in category 2 and category 4), we excluded these binary choices from the choice probability calculation. For premarking predictions, the probability of a correct choice was calculated over the summed activations of all features from a feature set given a premarker and the constant cue.

#### 2.2.4 Results and discussion

**Fig. 2:**
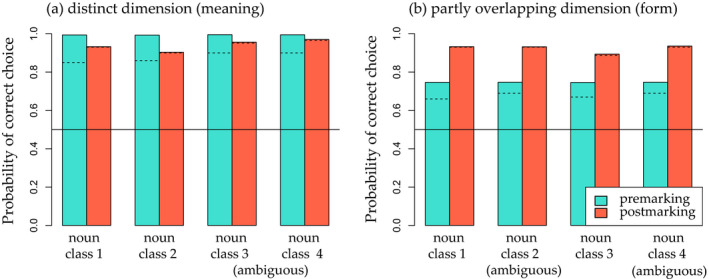
Probabilities of correct categorization (a) on the distinct dimension and (b) on the partly overlapping dimension after premarking training and after postmarking training to asymptote (1,600 trials) in Simulation 1. Blue bars show the probability of correctly choosing a feature set given a premarker and the constant cue. Orange bars show the probability of correctly choosing a postmarker given a feature set and the constant cue. Baseline performance, which assumes a completely naive model making a random choice, is marked by the horizontal line. The dashed lines show probabilities of correct choice after the same amount of training trials as in the behavioral experiment (412 trials). See Table 3 for all possible feature combinations.

The results of our simulation suggest that linear order of marking affects only categories that share overlapping features. Fig.2 summarizes the probabilities of correct categorization for all categories and by premarking and postmarking training. Categorization performance for overlapping feature sets (e.g., for Noun class 1, {D1form, O1form, O2form}) was higher after postmarking than after premarking (Fig. 2b). In turn, for distinct feature sets (i.e., for Noun class 1, {D1meaning}), we observed a small premarking advantage (Fig. 2a).

**Fig. 3:**
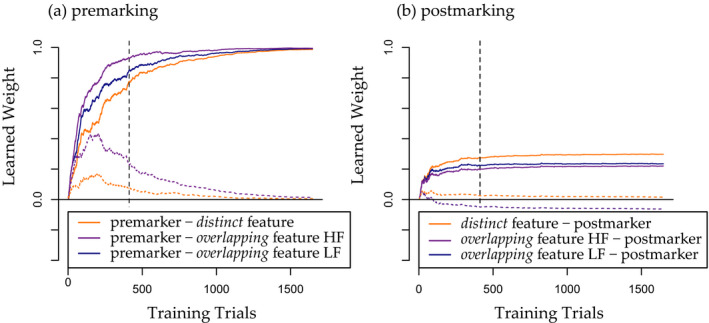
Learned weights of Noun class 1 in Simulation 1 (a) between premarkers (i.e., marker 1) and item features (i.e., {D1form, O1form, O2form, D1meaning}) and (b) item features and postmarkers (i.e., also marker 1). Orange lines show the weight between a distinct feature (i.e., D1form or D1meaning) and a marker, blue lines the weight between a low‐frequency (LF) overlapping feature (i.e., O2form; LF because occurring in two noun classes) and a marker, and violet lines the weight between a high‐frequency (HF) overlapping feature (i.e., O1form; HF because occurring in three noun classes) and a marker. Solid lines mark the correct features and dotted lines the features of the wrong Noun class 2. The vertical dashed lines show 412 training trials, as administered in the behavioral experiment.

An inspection of the learned weights of both models offers insight into the learning processes leading to these results. Weight development clearly differed between premarking and postmarking training (see Fig. 3) and shows that while postmarking seems to rely mainly on informativity, premarking seems to rely more on frequency. Before reaching asymptote, the premarking weights are ordered by frequency, with the least frequent, distinct features being learned slowest, the lower‐frequency overlapping features (that appear in less categories) being learned at medium speed, and the higher‐frequency overlapping features (that appear in more categories) being learned fastest (see Fig. 3a). This is in line with the idea that learning in a divergent learning relation is mainly driven by frequency (Ramscar, 2013). In our premarking model, the noun features compete with each other as *outcomes* for the small set of marker *cues* and learning does indeed seem to be driven by the frequency of the noun features. During postmarking training, the weights are arranged in the reverse order, with the least frequent but most informative distinct features being learned fastest (see Fig. 3b). In this case, the noun features are competing as *cues* for the marker *outcomes* in a convergent learning relation. Cue competition is therefore helping to dissociate the less informative overlapping features and concentrate on the more informative distinct features. As a consequence, less misclassification of feature sets with overlapping features (e.g., {D3form, O1form, O3form}) occurred in the postmarking model as compared to the premarking model, which was advantageous in the partly overlapping dimension but not in the distinct dimension (e.g., feature set {D1meaning}).

Note that the prominent difference in overall magnitude of premarking and postmarking weights emerges due to the restriction of the possible outcome activation in the learning algorithm to 1. As the outcome activation equals the summed weights of cues in a set to an outcome and as cue sets are larger in postmarking (e.g., {D1meaning, D1form, O1form, O2form, #o, k, ∫, a, m#, dog, constant}) than in premarking (e.g., {marker1, constant}), single weights in postmarking are much lower.

To be able to observe the complete learning process over time, we trained the models until weights between markers and noun features had reached asymptote. Clearly, in simple models like these, simulated learning time cannot be taken to predict actual learning in our participants. However, since the learning rates were held constant in the models, these training times can still play an informative role for the purpose of model comparison. Accordingly, we inspected the models’ performance at an earlier stage in which the number of simulated training trials equaled the number of empirical training trials in the behavioral experiment. This revealed that the probabilities of correct choice in both models and both category dimensions were already relatively constant at this earlier stage of training (see Figs. 2 and 3).

Finally, the ambiguity manipulation did almost have no effect on the models' categorization performance. While premarking was not at all affected, the postmarking models showed a very small effect with a slightly higher probability to choose the correct postmarker for items of ambiguous categories. This effect probably originates in the higher frequency of ambiguous features, which therefore get dissociated more strongly from competing category markers.

To assess the significance of the observed results, we performed two randomization tests comparing mean differences between the premarking and postmarking models in the reported simulation and in 1,000 random baseline simulations (see, e.g., Edgington & Onghena, 2007, and Appendix A). The first randomization test performed on the overlapping category evaluation showed that the difference between the means of the postmarking and premarking model significantly differed between the reported simulation and the random baseline simulations, with a postmarking advantage only appearing in the reported simulation but not in the random simulations (0.226 vs. −0.019, *p* = .001). The second randomization test performed on the distinct category evaluation showed that the result of the reported simulation was not significantly different from the baseline models, confirming the absence of a difference between premarking and postmarking regarding the evaluation of distinct category learning (−0.019 vs. −0.040, *p* < .192).

In sum, on top of a postmarking advantage in line with previous findings (Nixon, 2020; Ramscar, 2013; Ramscar et al., 2010; St Clair et al., 2009), this simulation suggests an interaction effect with category structure: Whenever frequency and informativity coincide, such as in learning of the distinct feature sets, premarking and postmarking training lead to similar categorization performance; only if informativity does not parallel frequency, postmarking training leads to an advantage for categorization supported by the mechanism of cue competition. The outcome of our simulation supports our first hypothesis that the postmarking advantage for learning categories does not generalize to categories which are perceived as distinct from each other. Besides this direct influence of linear marking order on discriminating the marked categories (noun class), we assume that it also has an indirect influence on learning subordinate category contrasts (noun meaning), which we explore in the following, second simulation.

### 2.3 Simulation 2: Linear order and levels of abstraction

Simulation 2 investigates the influence that linear marking order has beyond the directly marked level, in this case, noun class. In particular, it simulates the way that linear order at a super‐ordinate level (noun class) influences learning of subordinate categories (noun meanings).

Learning categories at different levels of abstraction, in this case, noun class and noun meaning, are clearly distinct tasks: While noun class learning involves associating a grammatical marker with a noun and its associated features, noun meaning learning involves associating a noun with items or events in the world. Although noun class markers are hence not *directly* involved in noun meaning learning, super‐ordinate category markers may have an *indirect* influence on subordinate category learning via their hierarchical connection. Specifically, premarkers, such as gendered articles, might lead to a facilitation of subordinate category discrimination by reducing uncertainty about items that follow them, such as nouns (Arnon & Ramscar, 2012; Ramscar, 2013) and their associated features. Accordingly, the noun class markers in our artificial language can be expected to serve to reduce uncertainty about the nouns and noun meaning pictures that will follow them in the behavioral experiment (see Section 3) in the same way, a process that this simulation seeks to model explicitly.

Technically, uncertainty reduction can be seen as a gradual reduction of the size of a set of expected outcomes that progresses as new information is received, with the set of expected outcomes itself being a function of prior learning. Accordingly, learners that have already acquired some form of hierarchical category structure might already expect a specific noun class—and thus a specific subset of nouns and noun meanings—after hearing a noun class premarker. This (implicit) set size reduction is important for the discrimination process because the updating mechanism of the error‐driven learning rule considers positive and negative evidence: After every learning event not only weights to present outcomes are adjusted but also weights to absent outcomes (third case of Eq. 2 in Section 2.2.1). This mechanism can therefore differentiate between cues that appear only with specific outcomes—informative cues—and cues that appear with many different outcomes—less informative cues. As the size of learning networks increases, it becomes more likely that cues occur with many different outcomes. Therefore, in larger networks, individual cues are less likely to be informative about specific outcomes. The size of the set in which the discrimination problem needs to be solved can thus be expected to directly influence how cue sets are associated with outcomes.

**Fig. 4:**
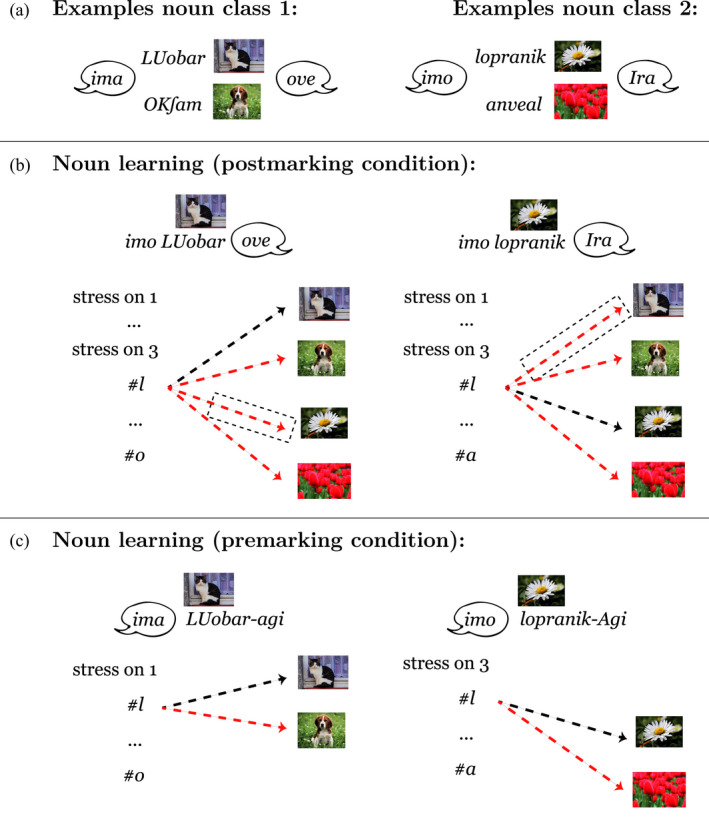
Illustration of the difference between learning to discriminate subordinate categories, here artificial nouns, with (b) postmarking or (c) premarking. (a) shows example nouns from two noun classes, with their associated premarkers and postmarkers (see Table 2). In postmarking (b) discrimination is performed *across* noun classes, which can lead to dissociation (red dashed line in black dashed box) of features relevant for the noun discrimination but overlapping between classes, for example, the first sound of a noun *#l*. Noun class premarkers (c) can reduce uncertainty about following items such that discrimination sis performed *within* a noun class.

Accordingly, if noun discrimination was only performed within and not across noun classes in our artificial language, the discrimination process would not be influenced by the nouns from other noun classes. The example in Fig.4 illustrates this idea. In our artificial category system, nouns with similar features occur in different noun classes. For example, some animal and plant nouns start with the sound *l* or *k*.^6^ When trying to solve the noun discrimination problem *across* noun classes (i.e., in the set of all nouns of all noun classes), features that discriminate nouns *within* a noun class would be dissociated as cues to specific objects of one noun class, when these features are shared with nouns from other noun classes, as depicted in Fig.4b. However, if the set size is reduced (e.g., by premarking), as shown in Fig.4c, also features that might be shared with other noun classes will be informative for the noun discrimination within a noun class and will not be dissociated.

The second simulation thus modeled the learning of noun–object associations in two ways: (a) the postmarking model was trained on the full set of nouns in one run and (b) the premarking model was trained separately on each noun class including only the respective subset of nouns; after training, we then merged the results of the separate premarking runs. This manipulation was based on the assumption that the perceived set size on the subordinate level is only reduced in the premarking condition but not in the postmarking condition.

After training, both models were tested on how well they could discriminate nouns within noun classes. Crucially, besides the set size difference during training, all other variables were kept the same between the premarking and the postmarking models: the number of noun–object events, the employed cue and outcome representations, and the linear order of noun and object representations. Regarding linear order of the noun and object representations, we considered the perceived order in the behavioral experiment (see Section 3). There, nouns and images of objects were presented at the same time (i.e., both follow immediately after the premarker, see Fig.6). However, under the assumption that acoustic noun processing generally precedes visual object processing (e.g., Jaśkowski, Jaroszyk, & Hojan‐Jezierska, 1990), we coded noun features as cues and noun meanings as outcomes in both models.

#### 2.3.1 Training

The noun stimuli used in this simulation were the same as used in the category learning simulation (Simulation 1, see Table 1). Both the premarking and the postmarking models were trained with noun form features as cues and objects as outcomes, for example:

{D1form, O1form, O2form, #o, k, ∫, a, m#, constant} → dog

While the postmarking model was trained on all nouns at the same time, the premarking model was trained separately on the nouns of every noun class, assuming that only a premarker can reduce uncertainty about possibly following nouns and objects. However, during the first quarter of training also the premarking model was trained on the full set of nouns because we assumed that premarker–object and premarker–noun associations first had to be learned to perform uncertainty reduction.

Note that we assume in this simulation that premarkers reduce the size of the set of nouns and objects associated with their meaning, thus cues *and* outcomes in the noun learning task. However, theoretically, only the reduction of the outcome set, thus of the objects, matters for the learning process because the discriminative learning algorithm in Eq. 2 updates weights to absent outcomes but not weights from absent cues.

Finally, as defined in the artificial language, also in this simulation noun frequencies within every noun class followed an exponential distribution. Learning parameters were set equally to the category learning simulation, and also here, a constant cue was added to every cue set.

#### 2.3.2 Model evaluation

To test the noun learning performance of the premarking and postmarking model, a noun feature set was shown to the model and the activation of the target object and competitor objects was calculated after the model had been trained to asymptote. In the postmarking model, all other objects were counted as competitors and in the premarking model only competitors within a noun class were considered. These activations were then normalized first with a rectified linear unit to correct for negative activations and then with the Luce choice rule to estimate the probability of a correct choice as in the category learning simulation. In the noun learning simulation, there was no problem of differing baselines between the premarking and postmarking models. Therefore, the probability *P_c_* of choosing the correct outcome *x* was calculated directly over the whole set of choice alternatives *O* and was not averaged over all possible pairs of target and competitors:

(5) $$P_{c}\left(x\right)=\frac{\text{ReLU}(\text{act}(x))}{\sum_{y\in 0}\left(\text{ReLU}(\text{act}(y)\right)}$$

#### 2.3.3 Results and discussion

**Fig. 5:**
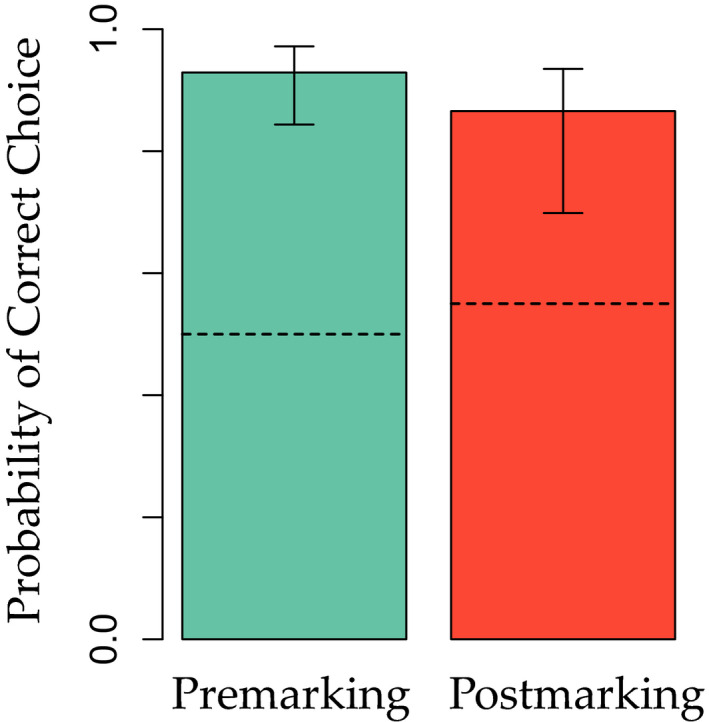
Median probability of choosing the target object in the noun learning simulation (Simulation 2) after weights of frequent noun features to objects have reached asymptote. Error bars show the interquartile ranges (i.e., 25%−75% of data). Dashed lines show median probability of choosing the target after the same amount of training trials as in the behavioral experiment (412 trials).

In the noun learning simulation, nouns in the premarking model were associated stronger to their target object than in the postmarking model, as illustrated in Fig.5. This suggests that optimization within smaller sets of nouns performs better than optimization in larger sets, which seems reasonable as in larger sets more random variation will lead to more noise during the learning process.

To reach asymptote, these models needed to be trained longer than in Simulation 1, due to the larger number of outcomes in this simulation. For the same reason, the premarking advantage also took longer to arise than the postmarking advantage in Simulation 1. We also inspected learning after the same number of trials as in the behavioral experiment. At this earlier point in training, the premarking advantage was still absent and overall the probability of correct choice was significantly lower in both the premarking and postmarking models.

To assess the significance of the observed premarking advantage, we performed a randomization test comparing mean differences between the premarking and postmarking models in the reported simulation and 1,000 random baseline simulations in which the outcomes in the training data were randomly shuffled (see Appendix A). The results of this randomization test indicated that the premarking advantage was significantly higher than in the baseline simulations with randomized outcomes (0.088 vs. −0.001; *p* < .001). This suggests that our reported simulation results were not due to random associations between single cues and outcomes.

Simulations 1 and 2 explored the generalizability of the postmarking advantage for learning categories using an error‐driven, discriminative learning mechanism. Simulation 1 showed that the postmarking advantage may not generalize to distinctly structured categories, and Simulation 2 showed that the postmarking advantage may not generalize to levels of abstraction subordinate to the marked category contrast. In addition, Simulation 2 suggests that premarking can facilitate discrimination by focusing the optimization problem on a smaller set of items. Regarding the underlying mechanisms, we found that cue competition determines when postmarking has an advantage in the marked domain (when item features overlap), and the global nature of the error‐driven learning process results in an advantage of super‐ordinate premarking for subordinate categories (because premarking can reduce the set size for the discrimination process). These findings form concrete and testable predictions for human learners when presented with the same artificial language. In the following section, we present the results of an artificial language learning study which tested these predictions on human learners.

## 3 Behavioral experiment

In an artificial language learning task using the same artificial language as in the simulations, we tested also linear order effects in differently structured categories and at different levels of abstraction. Participants were asked to listen to sentences in an artificial language, which was the same as the one presented in Section 2.1. In the sentences, the type of noun class marking was manipulated, a participant was either presented with only the premarking or only the postmarking variant of the artificial language. After the training phase, we tested to what extent participants had implicitly learned to categorize nouns into different noun classes along two dimensions (one distinct and one overlapping) and to associate nouns with object images. In this way, we could address both of our main questions in the behavioral experiment: First, we could test how category structure and linear order interact in learning by comparing the effect of linear order in learning the overlapping and distinct noun categories (which were combined to form four noun classes, see Table 2). Second, we could test the interaction of linear order with level of abstraction by investigating how marking order affected the learning of the noun meanings, a learning process which is subordinate to the noun class categorization.

The behavioral experiment was designed as a multi‐modal artificial language learning task in which we tested participants' ability to generalize implicitly learned category knowledge to new items (as in, e.g., Mirković & Gaskell, 2016). Participants were trained by listening to sentences while seeing corresponding images on the screen. To ensure that participants watched the screen, we tracked their gaze during the whole experiment. A training and test trial would only start when the participant had fixated the fixation cross for 500 ms without interruption.

We expected to observe an effect of linear marking order on how well noun classes were learned, in line with previous studies (e.g., Ramscar, 2013; St Clair et al., 2009). Moreover, based on our two simulations, we expected two interaction effects: First, a postmarking advantage is only for the overlapping form categories, but not for the distinct meaning categories; second, a premarking advantage is for noun learning, because the discriminability of subordinate categories (noun meanings) will increase by premarking of super‐ordinate categories (noun class).

### 3.1 Participants

After excluding two participants because their gaze behavior indicated that they did not look at the pictures on the screen, we analyzed data of 30 participants from the Groningen area (22 females and 10 males) who had participated for 8 Euro in this 1‐hr experiment (*M*_age_: 22.5, range: 18−28). All participants were Dutch native speakers. Eight of the participants were raised bilingually: six with Frisian, one with German, and one with Spanish.

### 3.2 Training stimuli

For training, the 32 imaginary nouns (50% two‐syllabic and 50% three‐syllabic) summarized in Table 1 were used. They were built into sentences according to the rules of the artificial language and recorded by a female speaker, who read them according to German orthographic rules and following the stress patterns specified for each noun class. A participant was either trained on the premarking or on the postmarking variant. The presentation frequency was modulated across items in each noun class fitting an exponential distribution (frequencies: 32, 23, 16, 11, 8, 6, 4, and 3).

**Fig. 6:**
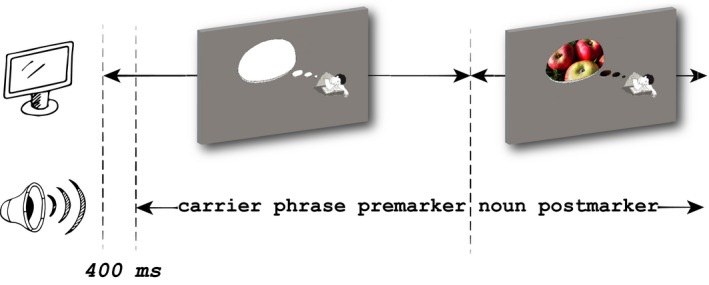
Sample training trial of the behavioral experiment. The image on the left depicts the sentence context matching the carrier phrase (he is dreaming of …), and the image on the right shows the context image with the noun meaning (apples) included.

For every presentation instance of a noun, a different photograph of the denoted object was shown (farm animals, flower plants, or random objects), integrated in a context image matching the carrier phrase (see Fig. 6). The images were chosen to produce high variation in background, color, image section, and number of items. Two context images matching the two carrier phrases (one version shown in Fig. 6) were combined evenly with instances of every noun and frequency subcategory. To eliminate bias for objects or categories, a different mapping between images and nouns was used for half of the participants. This yielded four experimental conditions: *premarking Version 1*, *premarking Version 2*, *postmarking Version 1*, and *postmarking Version 2*.

The order of the sentence stimuli was pseudo‐randomized: To assure that low‐frequency items would not appear too early, at first, 28 items were randomly picked from the four higher‐frequency categories of every noun class (112 items in total) and shuffled. The remaining 300 items were then randomized and appended. This order of sentences was maintained for all participants and conditions.

### 3.3 Test stimuli

**Fig. 7:**
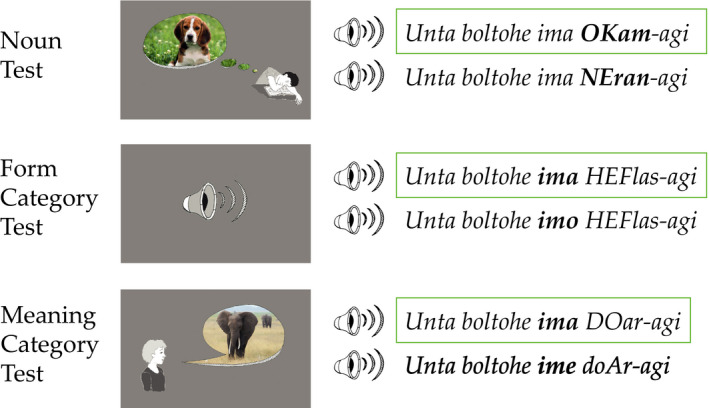
Sample test trials for the Noun, Form Category, and Meaning Category Test in the *premarking variant* (i.e., premarker varying with noun class and unspecific postmarker *agi*). Syllable stress is marked by capitals. The green boxes signal the correct answer options.

We tested learning of the distinct and overlapping categories as well as learning of the noun items in three two‐alternative forced‐choice tasks with two auditorily presented full sentences as choice alternatives. Fig.7 illustrates the three tasks. The participants were instructed to make a grammaticality judgment on these two alternatives by deciding which of the sentences sounded more correct. All test items were presented in the same randomized order in all four conditions. The three types of stimulus sets are presented below.

#### 3.3.1 Noun Test

All stimuli from training (see Tables 1 and 4) were presented with 50% old images and 50% new images (depicting an unseen token of the trained referent, e.g., an unseen dog species), which yielded 32 trials. Answer options were either a training sentence that matched the depicted referent or a training sentence that referred to another item within the same noun class (e.g., a cat instead of a dog). Note that the stress pattern was the same in both choice alternatives conforming to the specific noun class.

**Table 4:** The training objects of the behavioral experiment

Animals	Plants	Random 1	Random 2	Frequency
Dog	Rose	Car	Airplane	32
Cat	Sunflower	Chair	Shelf	23
Chicken	Tulip	Banana	Apple	16
Horse	Orchid	Lake	Mountain	11
Pig	Dandelion	Sewing machine	Flat iron	8
Mouse	Poppy	Kite	Ball	6
Sheep	Daisy	Fence	Umbrella	4
Rabbit	Forgetmenot	Foot	Ear	3

#### 3.3.2 Form Category Test

We created eight new nouns for every noun class (see Table B1) and incorporated them into two kinds of sentences presented as answer options (yielding in total 32 trials); in correct answer options, marking matched the stress pattern of the noun and in wrong answer options, markers of another stress pattern were presented. Importantly, all images were replaced by a loudspeaker icon, so that participants would only base their grammatical judgment on acoustic cues.

#### 3.3.3 Meaning Category Test

For each of the two semantically consistent noun classes, images of six new objects (farm animals or flower plants as in the training set) and six related new objects (safari animals or flowerless plants) were presented with new nouns embedded into sentences. In correct answer options, marking and stress were consistent with the class of the noun, and in wrong options, marking and stress were consistent with another noun class. For the two semantically random noun classes, six new objects and nouns were presented in a similar way. This yielded 36 trials in total (see Tables B1 and B2).

### 3.4 Procedure

The participants were trained and tested in a quiet room in which they listened to the recorded sentences with headphones, seated in front of a computer screen. To limit eye strain, all images appeared on a gray background. Participants were instructed in written form that they would learn a language from a fictive planet and that they should just listen to the playback sentences and watch the images on the screen attentively. They were kept naive regarding any information about the language and sentence structure and regarding details about the tests following the training. The training block was split into four blocks of 103 trials.

In training trials, first, the empty context image appeared, followed by the carrier phrase after 400 ms (see Fig. 6). The frame in the context image stayed empty for the length of the carrier phrase and the premarker and was then filled at onset of the noun. The mean length of a sentence recording was 2,487 ms (range: 2,247–2,953 ms), the mean length of a trial was therefore 2,887 ms. After every trial, a blank screen was shown for 100 ms, followed by a central fixation cross for 500 ms. Although the noun and object image were shown at the same time, to make sure that semantic and form categories could be premarked and postmarked, we assumed that the object image was processed slightly before the noun, based on evidence that visual stimuli are processed faster than acoustic stimuli (e.g., Jaśkowski et al., 1990). This matters for our assumption that during premarking the possible number of objects as referents for a noun is reduced to the members of the noun class, as depicted in Fig.4.

The test block started with the Noun Test, followed by the Form Category Test and then the Meaning Category Test. Between every test type, participants had the opportunity to take a self‐paced break.

In forced‐choice trials, context and object image were presented simultaneously and two answer options were played after each other. The participants had to press one of the two keys on a keyboard to indicate which sentence sounded more correct. To make the mapping between the presented answer options and the two keys for the answer options more clear, an icon on the lower left of the screen lighted up during the presentation of the first sentence and an icon on the lower right when the second sentence was presented. In half of the trials, the correct sentence was played first and in the other half, the incorrect sentence. After both sentences had been presented (again around 2,487 ms per sentence), the participant could press one of the two answer buttons in a time window of 2,000 ms. In the Form Category Test, the context and object image were replaced with a loudspeaker icon.

### 3.5 Results

**Fig. 8:**
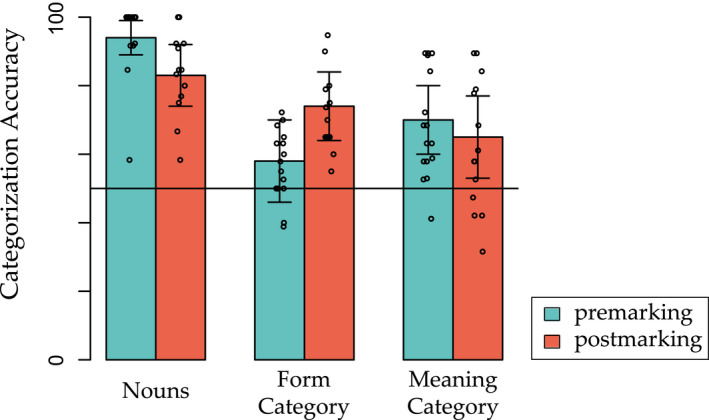
Model estimates (excluding random effects, CI ± 1 *SE*, inverse logit transformed; using R package itsadug) of accuracy in the Noun Test, Form Category Test, and Meaning Category Test for *correct answer options preceding wrong answer options* in the forced‐choice task (see results for wrong answer options preceding correct answer options in Fig. C1). Dots represent the actual data, namely mean accuracies by participant.

Fig.8 shows the result of the Noun Test, the Form Category Test, and the Meaning Category Test in the behavioral experiment. In the Noun Test, higher accuracies were observed after premarking training, but in the Form Category Test higher accuracies were observed after postmarking training. No accuracy difference was found in the Meaning Category Test. These observations are all in line with the predictions of our simulations.

The accuracy data of the forced choice tests were analyzed with generalized additive mixed‐effects regression modeling (Wood, 2011, 2017), which is a nonlinear regression method that allows us to include nonlinear effects of frequency and nonlinear random effects. We built two models predicting accuracy, one comparing the form and meaning tests and one investigating the noun test (see Supporting Information for code and output). The models had been constructed in an iterative backward fitting procedure using model comparison with χ^2^ tests and evaluation of Akaike's information criterion (AIC; Akaike, 2011), implemented in the R package “itsadug” (van Rij, Wieling, Baayen, & van Rijn, 2017). We did not analyze reaction time data as the auditory forced‐choice task resulted in a forced delay of participants' reactions.

The first model investigated the hypothesis about how linear marking order interacts with category structure by contrasting the data from the Form and Meaning Category Test including the predictors *Marking* (premarking/postmarking), *Task* (form/meaning), and *Target Position* in the forced‐choice tasks (correct sentence played first/second). The random effects structure included a random intercept for items (pairing of target sentence and picture) and participants, and random slopes for Task and Target Position by participants.

**Fig. C1:**
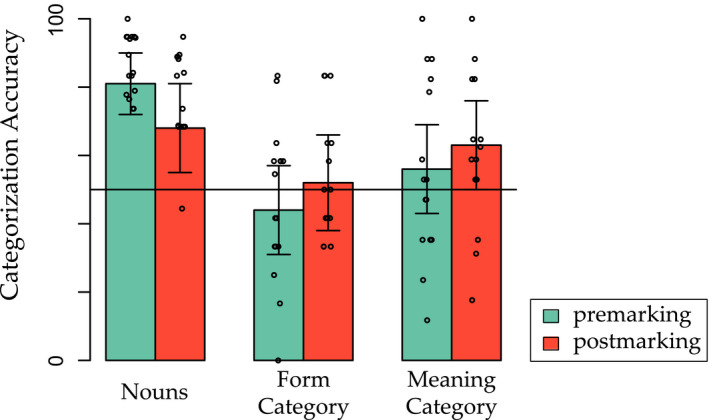
Model estimates (excluding random effects, CI ± 1 *SE*, inverse logit transformed; using R package itsadug) of accuracy in the Noun Test, Form Category Test, and Meaning Category Test models for wrong answer options preceding correct answer options in the forced choice task. Dots represent the actual data, namely mean accuracies by participant. The only significant difference between premarking and postmarking was an advantage of premarking for noun learning.

The best‐fitting model comparing the form and meaning task included a significant three‐way interaction of Marking, Task, and Target Position (χ^2^(1) = 2.213, *p* = .035; AIC difference: −1.78). We found a significant postmarking advantage for learning the form categories. In the Form Category Test, accuracy after premarking training was lower than after postmarking training (*β*_Premarking_ = −0.73, *SE* = 0.36, *z*‐value = −2.00, *p* = .045; see Fig. 8). After postmarking training, accuracy was significantly above chance level (*β*_Intercept_ = 1.05, *SE* = 0.27, *z*‐value = 3.91, *p* < .001). However, this postmarking advantage was not present when correct answer options were presented second (*β*_2nd_ = −0.95, *SE* = 0.30, *z*‐value = −3.14, *p* = .002; see Fig. C1). Also, the Meaning Category Test did not show a postmarking advantage, with accuracy after premarking training being higher than in the Form Category Test (*β*_Premarking:Meaning_ = 0.98, *SE* = 0.44, *z*‐value = 2.22, *p* = .026), but not the accuracy after postmarking training (*β*_Meaning_ = −0.43, *SE* = 0.32, *z*‐value = −1.35).

To test the second question about how linear marking order interacts with levels of abstraction, we ran a separate model on the noun learning accuracy data. This allowed us to include predictors unique to the Noun Test. We tested the predictors Marking, Target Position, *Stress* (on first/second/third syllable), *Frequency* (3, 4, 6, 8, 11, 16, 23, and 32) of nouns during training, and whether a picture in the test was new (*New*, levels: new/old). We included random intercepts for participants and items as well as a random slope for Target Position. The best‐fitting model showed main effects of the predictors Marking (χ^2^(1) = 3.761, *p* = .006; AIC difference: −0.83), Target Position (χ^2^(1) = 3.902, *p* = .005; AIC difference: −1.31), Stress (χ^2^(4) = 13.690, *p* < .001; AIC difference: −9.21), and Frequency (χ^2^(4) = 13.964, *p* < .001; AIC difference: −5.07). The predictor Marking showed a premarking advantage for the Noun Learning Test: After premarking training, accuracy was significantly higher than after postmarking training (*β*_Premarking_ = 0.92, *SE* = 0.34, *z*‐value = 2.75, *p* = .006). Moreover, when correct answer options were presented second in the forced‐choice task, accuracy was significantly lower than when correct answer options were presented first (*β*_2nd_ = −0.96, *SE* = 0.33, *z*‐value = −2.88, *p* = .004). For Stress, we observed that when nouns were stressed on the third syllable, they were learned less accurately than compared to nouns stressed on the second syllable (*β*_Stress3_ = −0.96, *SE* = 0.29, *z*‐value = 3.27, *p* = 0.001). Furthermore, accuracy increased linearly with increasing Frequency ($\chi_{\text{Frequency}}^{2}\left(1\right)=18.702,\;p<.001$).

Note that in both regression models, we did not find any difference in accuracy between the four noun classes and their ambiguity status, as also predicted in Simulation 1 (see Section 2.2.4).

### 3.6 Discussion

In the behavioral experiment, we found that, overall, learners were able to generalize learned category knowledge to new items exhibiting the features that were informative about the trained categories. Concerning our two hypotheses, we found evidence for interactions of linear order with both category structure and levels of abstraction. First, as in Simulation 1, we observed that linear marking order interacts with category structure. Postmarking facilitated learning the overlapping form categories more than premarkers (in line with Ramscar, 2013; St Clair et al., 2009) but, crucially, no facilitatory effect of postmarking was visible for learning the distinct meaning categories. While for discriminating noun classes by stress pattern, postmarking was advantageous, premarking and postmarking training led to a similar performance for discriminating noun classes by the meaning features. This suggests that although the postmarking advantage is an effect frequently found and cited in the literature, it does not generalize to every kind of category structure.

Second, our behavioral results show that this postmarking advantage does not generalize to categories at levels of abstraction subordinate to the postmarked category contrast. While we found that postmarking facilitates learning the super‐ordinate noun class categories, we found that premarking facilitates learning the subordinate noun categories. This effect is in line with the hypothesis based on Simulation 2 and previous evidence that premarking of super‐ordinate categories brings an advantage in learning subordinate categories. In Simulation 2, we assumed that learners could use the premarkers to reduce the discrimination process to a single noun class, which enhanced noun learning. As our learners already had category representations for the semantic categories prior to the experiment and probably quickly learned to associate premarkers with a semantic category, they could use premarkers to predict a subset of objects, for example *animals*. Subsequently, associating an unknown noun with an object within a noun class (e.g., a noun with a dog within the animal category) was then easier for the learners than across all noun classes, as suggested in Simulation 2.

Regarding the premarking advantage for noun learning, a next step could be to further investigate in what situations premarkers can reduce uncertainty about following information. In our behavioral study, premarkers were presumably used to reduce uncertainty about following information, in our case noun semantics in the object images, although this association was not separately trained before. Also Arnon and Ramscar's (2012) findings suggest that in an immersive learning situation it is possible to associate premarkers with familiar noun semantics fairly quickly such that they can be directly used to enhance learning artificial nouns' meanings in the same training session. This quick association was probably facilitated by previous knowledge of the learners about the objects and semantic categories in the experiment. In contrast, it might be more difficult to learn to associate premarkers with unknown objects, resulting in less uncertainty reduction and, thus, less facilitation for learning to associate nouns with these objects. A positive effect of premarking as we observe it here might therefore be restricted to specific learning situations, such as second language learning, or might take more time to emerge for completely naive learners.

Further predictors we found to influence learning of the noun meanings, here the subordinate category contrast, were noun frequency and stress. The facilitative effect of noun frequency shows that frequency of occurrence of a cue–outcome pair can lead to faster learning of an association, also when this factor should not be regarded on its own irrespective of other factors, as for example informativity (cf. Rescorla, 1988). Regarding the different stress patterns of the nouns, we observed that nouns with stress on the second syllable, a frequent stress pattern in Dutch, were learned better than nouns that were stressed on the first or last syllable, which are less common in Dutch. It seems that it was easier for our learners to link this familiar stress feature to a new word meaning than an unfamiliar stress pattern. This suggests that frequency of presentation during training can positively influence learning and that features that are infrequent in a native language might be harder to integrate into a new language system.

For form category learning and noun item learning, we furthermore found an effect of the order in which the answer options appeared in the forced‐choice task. We suspect that using a forced‐choice task with auditory instead of visual stimuli imposes a processing order and at the same time a processing limit without allowing for regressions. As a consequence, if the gap in time between two answer options is not big enough, the first answer option might still be processed when the second answer option is presented. In our study, we found that when correct answer options preceded wrong answer options in the test, accuracies were higher and therefore differences between the conditions were also more pronounced (compare results for correct answer option coming first in Fig. 8 and results for wrong answer option coming first in Fig. C1). In the Form Category Test and Noun Test, we therefore found a clear difference between marking conditions, with either postmarking (in the Form Category Test) or premarking (in the Noun Test) showing accuracies significantly above chance, when the correct answer option was presented first. When the wrong answer option was presented first, we found that accuracies in both marking conditions were overall lower (for noun recognition) or even at chance level (for form categories). We presume that wrong answer options are processed more slowly than correct answer options, given that learners had more exposure to the correct patterns during the training phase. Therefore, while for the correct answer options the short processing window of the first answer option might have been sufficient, it probably was not long enough for the wrong answer options. In turn, the resulting lack of processing of one answer option presumably impeded the comparison of the two answer options. We are not aware of many studies applying this kind of acoustic forced‐choice task, except for the related testing procedure of Arnon and Ramscar (2012). They did not report such an effect of answer option order; however, given that their task was overall easier than our task, we assume that the limited amount of processing for the first answer option did not lead to an effect in their study. While they tested the nouns presented during the training phase, we tested novel nouns and, in addition, our learners were exposed to a more complex category system with more feature dimensions. Overall, we suggest that the effect of the order of answer options in our study reflects an increased task difficulty which causes problems when wrong answer options are presented first in a restricted time window. Importantly, this pattern of results does not seem to suggest a general bias, as neither the first nor the second answer was preferred more over the other option.

Thus, to summarize, the interactions of marking order with category structure and levels of abstraction we observe in the behavioral experiment suggest that linear order effects such as the prominent postmarking advantage for category learning do not generalize to distinctly structured categories and to subordinate categories. Furthermore, we confirmed the previous finding of a premarking advantage for learning subordinate categories.

## 4 General discussion

This study sought to investigate the effects of premarking and postmarking on learning linguistic categories of different structures and at different levels of abstraction. In addition to offering a formal account of these effects, the findings of our investigation also offer insights into the functions that premarking and postmarking have in category learning.

Our manipulation of category structure in the behavioral study showed that the often cited postmarking advantage (e.g., Clark, 2001; Kuczaj, 1979; Ramscar, 2013; Ramscar et al., 2010; Slobin, 1973; St Clair et al., 2009) for learning categories does not generalize to distinctly structured categories. Only when categories are perceived to have overlapping/confusable features, they were more easily associated with postmarkers than with premarkers. Simulation 1 showed a similar effect and suggests that the convergent learning relation present during postmarking is particularly suitable to dissociate non‐discriminating features from a postmarker according to their informativity for the category contrast. In a divergent learning relation usually found during premarking, learning is more dependent on the frequency of markers and features, and less on the informativity of features for a marker and the connected category contrast. Whenever dissociation of uninformative features is not needed, as in the case of categories which are already perceived as distinct because they have already been formed, postmarking does not show this advantage. In that case, learning of the category contrast will proceed comparably in pre‐ and postmarking.

We conclude from these findings that postmarking has a functional role in learning to form *new* categories by providing distributional information in the linguistic input which can be directly used to build discriminative category representations as opposed to probabilistic category representations that are built from premarking (which is in line with previous evidence; see Ramscar, 2013; Ramscar et al., 2010).

As a second main finding, we found that while during postmarking training, categorization of the marked noun class categories was facilitated, categorization of the subordinate noun categories was inhibited. This corroborates the assumption that categories at different levels of abstraction stand in a trade‐off relation with each other as, depending on the task, contrasts at different levels might be relevant (Dye & Ramscar, 2009). Our second discriminative learning simulation shows how premarking can facilitate learning of subordinate categories. We assumed that premarkers can reduce uncertainty about following information when they are trained to predict, for example, following words or their meanings. Given this assumption, premarking probably leads to discrimination in a smaller set of nouns than postmarking, and Simulation 2 shows how discrimination in a smaller set can be more effective than in a larger set. Hence, premarking seems to have an important role in discriminative *processing* (i.e., uncertainty reduction), which, in turn, can facilitate learning by restricting the discrimination process to a specific set of cues and outcomes.

More generally, our findings contribute to a growing body of evidence that discriminative learning is not only influenced by how frequently a cue and an outcome co‐occur. While we do find a facilitatory effect of frequency for noun learning, we observed three crucial additional factors. First, learning can be influenced by the ratio between cues and outcomes. Second, when there are more cues than outcomes, learning might also strongly depend on the informativity of single cues for outcomes. Third, Simulation 2 suggests that learning success can be determined by the size of the set in which the discrimination problem needs to be solved.

### 4.1 Generalization to natural languages

Working with artificial languages always raises the question how they are representative of natural languages. Our noun class system partly resembles natural languages but is partly also too simplified. In natural languages, noun class can align with form features (e.g., as Hohlfeld, 2006, suggests, in German gender) or semantic features (e.g., noun classes in Swahili) as in our artificial language. However, categories in natural language often do not align directly with other perceptional or conceptual categories. German gender, for example, partly aligns with semantic features but partly also violates these rules. While the partial alignment in features does facilitate learning in general, the highly frequent outliers (e.g., fork, knive, and spoon have three different genders in German) are better learned in a process of discrimination. Thus, in natural languages, it becomes even more apparent that categories are not merely a taxonomic but a discriminative system which probably requires mechanisms of clustering by both similarity and discrimination.

We should, however, sound a note of caution when it comes to directly generalizing the results from a restricted experimental setup to the full complexity of natural language learning. As our results show, linear order is of importance only when categories show overlap that leads to a confusion of item features that are highly frequent with item features that are informative about a category. Other factors that might influence effects of linear order in natural languages are, for example, the distance between a word and its category marker (as, e.g., in longer agreement dependencies^7^) or the within‐category item distribution. Given the complexity of natural languages, it follows that we clearly need variations of this experiment to better understand exactly which category characteristics apart from linear order influence learning, and how they influence it. For example, tests of other modalities, of different relations of category contrasts, and a comparison of children and adults could all be informative in this regard.

### 4.2 Linear order in natural language learning situations

To generalize our findings in an artificial language learning situation, it is also important to consider how linear order of stimuli can be established in natural language learning situations. In our case, the order of markers and noun features was set artificially to exactly lead to a pre‐ and postmarking situation for auditory and visual features. In the domain of auditorily presented speech, this order comes naturally, but in the domain of visually presented semantics, we had to force this order. While objects are usually constantly present in a visual scene, our object images appeared only after the premarker had been auditorily presented. It is, however, possible that in a natural language learning situation, for example, when a child learns the names of toys, real premarking might be rather rare, even in premarking languages, because, for example, the child has the possibility to play and see the objects before any speech is uttered by a parent. On the other hand, there might be several factors which modulate the operationalization of sequence in a natural learning situation, for example, mechanisms such as joint attention or task effects. Also note that we restrict our reasoning here to the learning of concrete nouns with directly accessible semantics and do not consider abstract noun learning. Crucially, the temporal dynamics of a natural learning situation are probably dependent on multiple temporal cues beyond word order.

### 4.3 Generalization to language acquisition

Lastly, we would like to shortly discuss how our findings can be generalized to second and first language acquisition. Our manipulation of category structure assumed that general semantic categories, such as animals or plants, have already been learned by our adult participants who would therefore perceive them as distinct. We observed that the participants in our behavioral experiment could readily associate these categories with a new category marker, irrespective of whether it was a premarker or a postmarker. We suggest that this situation occurs frequently in second language learning when category systems of the first and second language are aligned. For example, an adult native English learner of French has already learned to discriminate dogs from other animals, and therefore just has to learn a new word form (“chien”) and map it to the already existing category representation. As no further dissociation of uninformative features is needed, postmarking will probably not bring an advantage for learning this new French category label. In turn, this also suggests that if we had tested infants on our artificial language, we might have found a postmarking advantage also for learning, for adults, the distinct semantic categories.

However, categories between different languages do not always align neatly. Much more often category systems differ significantly and many difficulties in second language learning stem from these differences. Frequently, second language learners have to learn new category contrast, which means that existing categories need to be split up into a more discriminative category system. This can be the case at different levels of abstraction, for example, when learning new sound contrasts such as tone (Nixon, 2020), when learning new grammatical contrasts such as noun class, or when learning new semantic contrasts such as new verb dimensions (Gullberg, 2009). In addition, category boundaries often need to be shifted to accommodate to a new category system of the new language (e.g., Boersma & Escudero, 2008). As opposed to situations in which previously learned categories can be reused, these situations require relearning of categories. We suspect that postmarking might facilitate this process. This would mean that we have to take linear order into account not only when it comes to newly building categorical perception, such as when infants learn their first language, but also when learned categorization preferences need to be overcome and restructured, such as when adults learn a new language with different category contrasts.

## 5 Conclusion

We have presented a unified account of linear order effects in different kinds of category systems that provides more insight in the role of categories and category marking systems in language. Given the present evidence and our interpretation within an expectation‐based learning account, we conclude that whenever category‐relevant features are in competition with irrelevant features, postmarking facilitates category formation. We suggest that this could be whenever categories have to be formed from a completely naive point of view, for example, in first‐language acquisition, or when category systems need to be reshaped, as often necessary in second language learning. When it comes to learning of subordinate categories, premarking shows its advantages, as it does not abstract away from features that are important for discrimination of more fine‐grained category contrasts, as it focuses the discrimination process on these subordinate category contrasts.

Our findings connect previous evidence about different characteristics of the learner input influencing the learning of linguistic categories within an expectation‐based theory of language learning. The interactions of linear order that we found with category structure and with levels of abstraction illustrate how linguistic categories need to be studied as part of a complex system of contrasts. These contrasts arise out of a need for discrimination, and depending on the situation, their importance shifts within and between levels of abstraction. We suggest that grammatical markers have an important role in balancing this system and guiding a learner to the contrasts that are relevant within a specific context.

### Open Research badges

This article has earned Open Data badge. Data is available at https://git.lwp.rug.nl/p251653/linear‐order‐and‐category‐structure.

## Notes

^1^ Henceforth, “item” is used to contrast the term “category.” However, on a potential lower level of abstraction, items can also form categories.

^2^ Depending on the length of the word stem, the last syllable could be the third or fourth syllable.

^3^ Stress can alter word meaning in Dutch: beDELen versus BEdelen.

^4^ The supplementary materials are available at https://git.lwp.rug.nl/p251653/linear‐order‐and‐category‐structure.

^5^ Note that the carrier phrases were not included in the simulations but were only used in the behavioral experiment to evoke a more natural learning situation with full sentences.

^6^ This kind of category structure can often be found among natural linguistic category systems. For example, in the German gender system, semantic features widely overlap between genders, such that, for instance, furniture items can be masculine (der Stuhl—the chair), feminine (die Lampe—the lamp), or neuter (das Sofa—the sofa).

^7^ For example, when gender postmarking appears on word *n* + 2, as in the following French example, where the gendered adjective “belles” marking gender on the noun “fleurs” appears after the verb “sont”: les *fleurs* sont *belles* (the flowers are beautiful).

## Appendix A Randomization tests performed for model evaluation

To assess the significance of the simulation results, we performed randomization tests (cf., e.g., Edgington & Onghena, 2007). Significance levels were computed by comparing mean differences of the probability of choosing the correct outcome between premarking and postmarking models in the reported target simulation meanDiff_t_ and *n* competitor simulations meanDiff_c_. For overlapping categories in Simulation 1 and in Simulation 2, competitor runs had to produce larger mean differences to challenge the target run:

(A1) $$p=\frac{\text{count}\left({\text{meanDiff}}_{c}\geq {\text{meanDiff}}_{t}\right)}{n}$$

For distinct categories in Simulation 1, competitor runs were counted when the mean differences were closer to zero than in the target run:

(A2) $$p=\frac{\text{count}\left(\left|{\text{meanDiff}}_{c}\right|\leq \left|{\text{meanDiff}}_{t}\right|\right)}{n}$$

## Appendix B Items used in the behavioral experiment

**Table B1:** The test nouns of the artificial language in the behavioral experiment

		Noun Class 1	Noun Class 2	Noun Class 3	Noun Class 4
Form Category Test		egadan	issater	vosshartin	meatok
		later	borlaw	nirmal	klertash
		tseglar	kambral	sormanir	senhar
		rishtar	atpos	biskrot	tunalig
		heflas	noemen	loer	resham
		seniter	impras	sutkar	naelis
		kurken	harmenat	elemor	liens
		doar	bukes	trame	rombad
Meaning Category Test	Close	heflas	wodnim		
		netad	tenglarol		
		ragun	bukes		
		bearel	sagav		
		seniter	fegon		
		ferutam	noemen		
	Far	kurken	kalmen		
		igaral	impras		
		eivomal	eanor		
		doar	daspal		
		ustiged	ograg		
		tamrog	harmenat		
	Random			loer	resham
				sutkar	naelis
				elemor	liens
				porfenet	grimnes
				trame	rombad
				guskar	lakafer

Note

In the Noun Test, all training nouns (see Table 1) were used as test stimuli. Note that while a few nouns orthographically look like Dutch words, they were read according to German orthographic rules and connected with a pre‐ and postmarker so that they did not sound like Dutch words.

**Table B2:** The test images of the behavioral experiment

		Animals	Plants	Random 1	Random 2
Meaning Category Test	Close	cow	snowlily		
		goat	lily		
		duck	crocus		
		donkey	daffodil		
		goose	hibiskus		
		hedgehog	buttercup		
	Far	elephant	fir		
		tiger	cactus		
		antelope	reed		
		giraffe	beech		
		zebra	grass		
		crocodile	boxwood		
	Random			cross	circle
				fire	water
				sun	snow
				drum	violin
				hat	gloves
				hammer	saw

Note

For the Noun Test, all training images were used as test stimuli. In all trials of the Form Category Test, a loudspeaker icon was shown instead of a picture. In the Meaning Category Test, test nouns either referred to a close or far category member, or to a random object for the two random noun classes.
